# 

**Published:** 1981

**Authors:** 


JULY/OCTOBER 1981
VOLUME 96 (iii/iv)
NUMBER 359/360

